# Determination of developmental and ripening stages of whole tomato fruit using portable infrared spectroscopy and Chemometrics

**DOI:** 10.1186/s12870-019-1852-5

**Published:** 2019-06-04

**Authors:** Paul Skolik, Camilo L. M. Morais, Francis L. Martin, Martin R. McAinsh

**Affiliations:** 1 Lancaster Environment Centre, Lancaster University, Bailrigg, Lancaster, LA1 4YQ UK; 2 School of Pharmacy and Biomedical Sciences, University of Central Lancashire, Preston, PR1 2HE UK

**Keywords:** Tomato, Development, Ripening, Crop biology, MIR spectroscopy, Chemometrics

## Abstract

**Background:**

Development and ripening of tomato (*Solanum lycopersicum*) fruit are important processes for the study of crop biology related to industrial horticulture. Versatile uses of tomato fruit lead to its harvest at various points of development from early maturity through to red ripe, traditionally indicated by parameters such as size, weight, colour, and internal composition, according to defined visual ‘grading’ schemes. Visual grading schemes however are subjective and thus objective classification of tomato fruit development and ripening are needed for ‘high-tech’ horticulture. To characterize the development and ripening processes in whole tomato fruit (cv. Moneymaker), a biospectroscopy approach is employed using compact portable ATR-FTIR spectroscopy coupled with chemometrics.

**Results:**

The developmental and ripening processes showed unique spectral profiles, which were acquired from the cuticle-cell wall complex of tomato fruit epidermis in vivo. Various components of the cuticle including Cutin, waxes, and phenolic compounds, among others, as well as from the underlying cell wall such as celluloses, pectin and lignin like compounds among others. Epidermal surface structures including cuticle and cell wall were significantly altered during the developmental process from immature green to mature green, as well as during the ripening process. Changes in the spectral fingerprint region (1800–900 cm^− 1^) were sufficient to identify nine developmental and six ripening stages with high accuracy using support vector machine (SVM) chemometrics.

**Conclusions:**

The non-destructive spectroscopic approach may therefore be especially useful for investigating in vivo biochemical changes occurring in fruit epidermis related to grades of tomato during development and ripening, for autonomous food production/supply chain applications.

**Electronic supplementary material:**

The online version of this article (10.1186/s12870-019-1852-5) contains supplementary material, which is available to authorized users.

## Background

Global food security relies on the combination of effective crop production, distribution, and utilization [1]. Crop production and distribution are both becoming increasingly challenging whilst population growth and changes in climate are leading to food shortages and malnutrition worldwide [2]. Conventional farming practices have struggled to increase the production of major crops worldwide [3]. Due to lack of available land for food production, it is expected that much of the increase in crop production will occur through higher yields, intensified cropping and a reduction of waste in the supply chain due crop loss to climate, pests, pathogens, as well as downstream consumer waste [4, 5]. Innovative solutions that maximize crop production and reduce waste are therefore of paramount importance to maintaining food security. While numerous approaches to aid with this are being developed, technology-based solutions to farming are frequently confounded by the large number of crop species (and cultivars) grown and the complexity of plant-environment interactions within crop production systems. Therefore, there is an urgent need for the development of novel approaches for improving our understanding of crop biology and the development of applied farming tools to maximize production, minimize losses and to improve pre- and post-harvest production and utilization.

Tomato (*Solanum lycopersicum*) is one of the most important crops globally valued at 124.6 billion US dollars annually [6] representing the largest sector of the fleshy fruit market [6, 7]. It is widely used as plant model due to its short generation time, and well-studied genetic, biochemical, and physiological properties [8]. Rich in beneficial phytochemicals, tomato fruit are delicate, develop and ripen quickly and are used at various stages of their development either whole, or for various processing purposes including canned goods, pastes, sauces, juices, etc. [9]. Each of these products require fruit at different stages of development or ripening ranging from early immature to red ripe fruit depending the number of days post anthesis (dpa) [9]. The development and ripening of tomato fruit both of which are parameters important to the horticultural industries influencing fruit quality and shelf life [7, 10]. The ability to accurately and non-destructively monitor changes occurring during tomato fruit development and ripening are therefore of utmost interest to both plant biologists and horticulturalists.

The plant epidermal layers and associated surface structures provide the plant-environment interface necessary for maintaining plant integrity, regulating fruit growth, and determining shelf life [10, 11]. Tomato fruit epidermis is composed of an integrated heterogeneous multi-layered matrix including the cuticle (cuticle proper and cuticular layer), cell wall, and epidermal cells [12]. These layers undergo extensive changes during fruit development and ripening. However, to date the molecular mechanisms involved and how these changes influence characteristics like morphology, texture, pathogen susceptibility and shelf life have not been elucidated fully [12]. In addition, it has been difficult to study the cuticle and cell wall separately due to the recalcitrant nature of these tissues [13]. Therefore, novel approaches to investigate plant surface structures are essential to determine how they contribute to the healthy growth and development, or appearance of abnormal conditions, in horticultural products. Furthermore, these approaches need to be translatable into practical field-based applications to have relevance to both fundamental plant biology studies and applied crop science. Although there are a number of analytical tools, traditionally used in the laboratory which might be suitable for field-based horticultural applications [14–16], the tools available to study plant surface structures non-destructively are limited.

Optical sensors based on light-matter interactions have been implicated as effective tools for the non-destructive monitoring of plant health and disease detection based on spectral signatures [14, 17]. Particularly mid-infrared (MIR) vibrational spectroscopy combined with chemometrics has been widely used as a bioanalytical tool that offers non-destructive analysis of most types of samples [18]. Vibrational spectroscopy, also known as surface techniques, typically probes the surface layers of samples to micrometre depths and, due to advancements in data analysis, can also be used to analyse complex heterogeneous biological samples, termed biospectroscopy. The unique spectrum of biological materials between 4000 and 400 cm^− 1^ (2.5–25 μm wavelengths), produced through light-matter interactions between the IR radiation and the sample, contains biochemically specific variables useful for biological applications [19]. Many biological materials absorb preferentially in the ‘fingerprint region’ (1800–900 cm^− 1^), therefore this region is often the spectral range selected for analysis [20]. The analysis of spectral data can be divided into exploratory and diagnostic analyses [21]. Exploratory data analysis includes data visualization, pattern recognition, and biomarker extraction [21, 22]. Examples of analysis models used for these purposes include unsupervised learning such as principal component analysis (PCA), and supervised methods such as linear discriminant analysis (LDA) [21]. Diagnostic data analysis aims at evaluating classifier performance for autonomous decision making. Various classifiers commonly used include LDA, support vector machine (SVM), naïve bayes, and artificial neural networks (ANN), each of which exhibits varying levels of model complexity. MIR spectroscopy together with specialized data analysis have been applied to address important horticultural issues including plant health monitoring, plant-environment interactions, disease detection, phenotyping, and taxonomic relationships [17, 23, 24]. However, the development of biospectroscopy-based bioanalytical approaches for crop science that allow plants to be studied both in the lab and in a field-environment is essential for its wider adoption as a horticultural tool [18].

Currently, portable Raman spectrometers, which can measure intact samples are more readily available than IR spectrometers with such a capability. Consequently, to date, progress towards the development of biospectroscopy-based bioanalytical approaches for the analysis of intact crops has been limited primarily to the use of Raman spectroscopy, although this technique has only been recently employed for whole sample analysis [25–28]. Several other techniques outside the MIR range such as near-IR (NIR), ultraviolet (UV) and Visible light, as well as hyperspectral analysis have been used to assess quality parameters in tomato [29–31]. However, few of these studies provide detailed biochemical insight into the changes occurring in vivo during development and ripening and have traditionally focused solely on classification performance or correlation between traditional quality parameters and spectral data [32]. Furthermore, the potentially small measurement area, as well as the higher energy of NIR, UV, visible, and Raman instruments, increases the light penetration depth into the sample over a very small area making it potentially difficult to obtain reliable biological information. MIR spectroscopy in contrast offers sampling modes with very well-defined measurement areas and light penetration depths [19], which permit biochemical investigations when combined with known chemical compositions of plant tissues under investigation [33–37]. Attenuated total reflection Fourier-transform infrared (ATR-FTIR) spectroscopy is one method with a very well-defined light penetration depth, where macro measurements over larger areas are possible [19]. In other fields, ATR-FTIR spectroscopy has proved exceptional at providing both biochemical insight into biological samples, as well as providing strong discriminating power in combination with classification models [20, 34]. This suggests a need to evaluate the use of Raman complementary methods such as reflectance spectroscopy including ATR-FTIR spectroscopy within crop science. In order to increase the capacity for spectroscopy-based methods to provide biochemical information as well as classification performance, it is imperative to assess complementary approaches aimed at developing multi-sensor platforms, which will be required for complex systems.

Tomato is widely used as a model system for studying cuticle, cell wall, and epidermis during fruit development and ripening. In the present study therefore, we apply a novel approach combining multivariate chemometrics for biomarker extraction and assessment of classification performance. Biomarker extraction as part of a two-tiered approach is aimed at studying the effects of development and ripening on the spectral signatures of tomato fruit. First, exploratory multivariate analysis in the form of PCA-LDA was used to extract tentative wavenumber biomarkers associated with differences in the nine developmental stages of tomato fruit from 4 to 36 dpa, and subsequently the six distinct ripening stages from mature green to red ripe tomato (approx. 34–55 dpa). Biochemical entities identified as biomarkers are explored. The second tier involves SVM classification of the nine developmental and six ripening stages, in order to determine the potential for autonomous grading of tomato fruit maturity and ripening stages based on MIR fingerprint spectra.

## Results

### Spectral analysis of tomato fruit Development

**Fig. 1:**
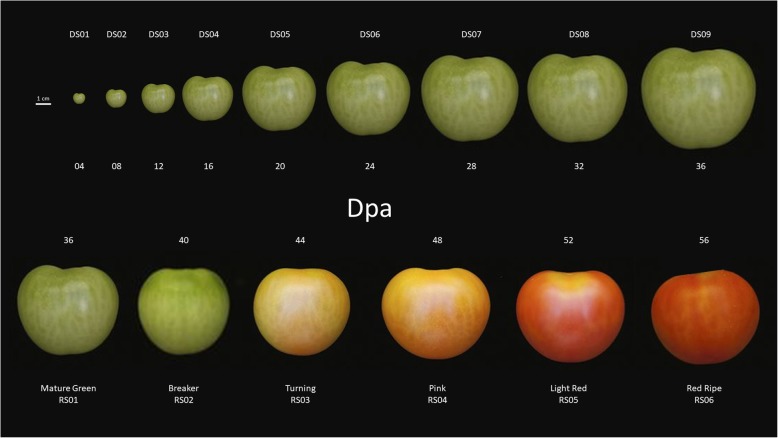
Tomato fruit of *Solanum lycopersicum* cv. Moneymaker: developmental (top) and ripening (bottom) stages used as individual groups for MIR ATR-FTIR spectral analysis; dpa (days post anthesis)

**Fig. 2:**
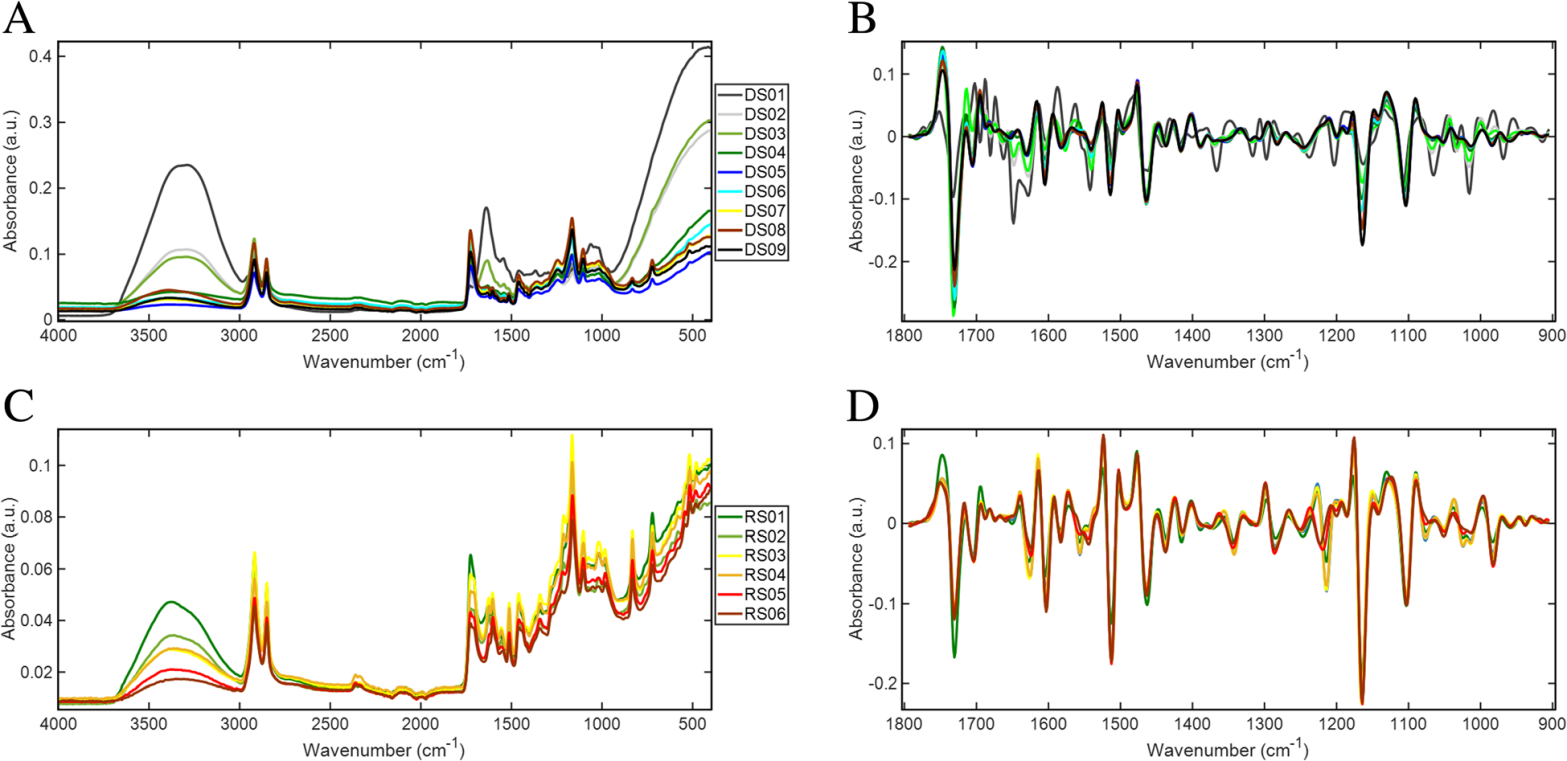
ATR-FTIR spectra as class means with raw and pre-processed spectra for development (**a** and **b**) and ripening (**c** and **d**)

Tomato fruit development and ripening were split into two distinct processes, as shown visually in Fig. 1. Spectra were acquired from each developmental timepoint including ripening. Figure 2 shows the class mean raw and pre-processed fingerprint spectra for the development (Fig. 2a and b) and ripening (Fig. 2c and d) processes. Figure 2 clearly shows that most sharp absorbance peaks are evident within the fingerprint region between 1800 and 900 cm^− 1^. This region holds most of the biochemical information pertaining to the samples and was therefore the focus of the investigation.

**Fig. 3:**
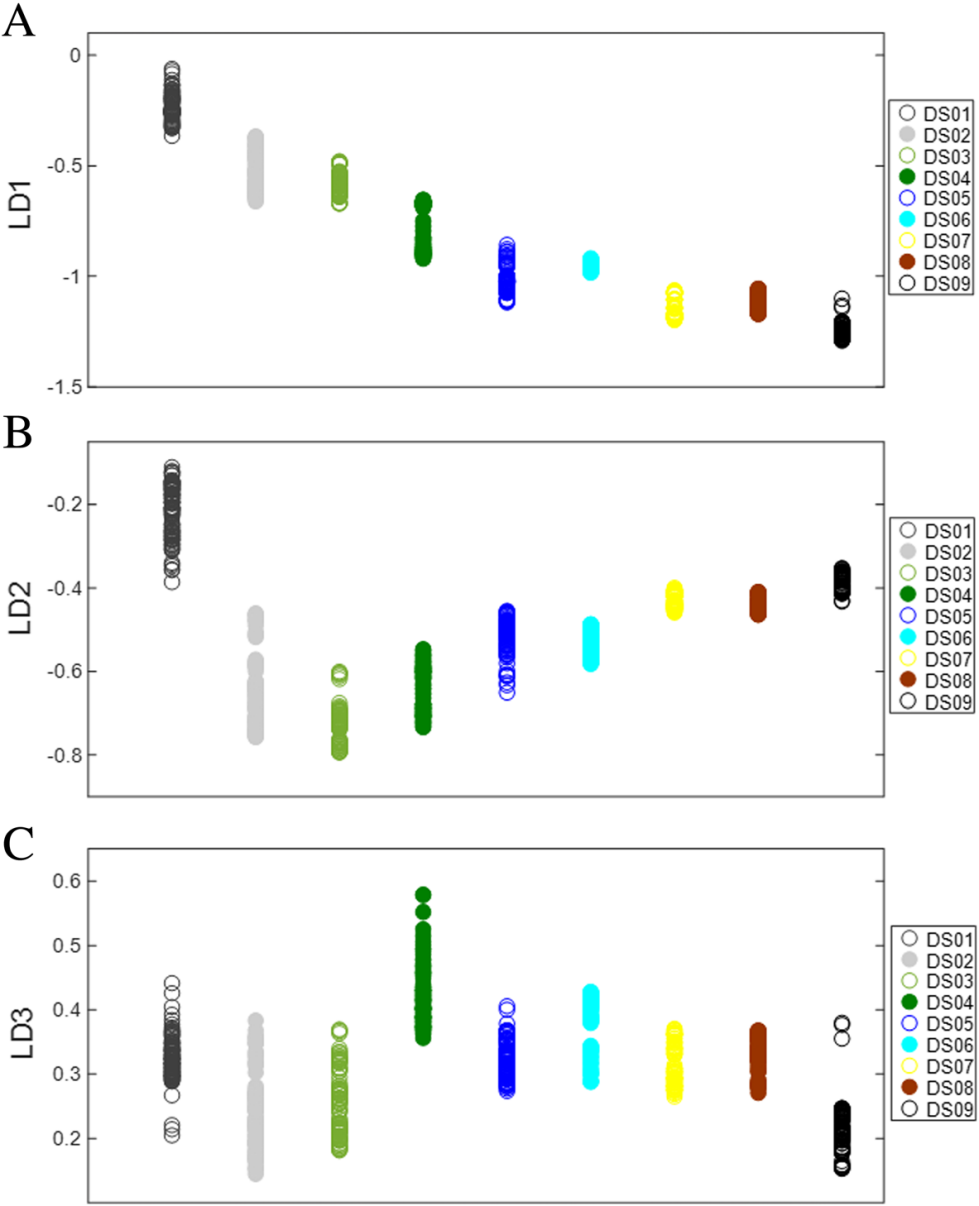
PCA-LDA 1-dimensional scores plots of tomato fruit developmental stages (DS01-DS09) along LD1 (**a**), LD2 (**b**), and LD3 (**c**)

Linear discriminant analysis effectively distinguishes tomato fruit development based on PCA factors. Figure 3 shows the three linear discriminants LD1, LD2, and LD3 respectively, based on LDA of PCA factors. Variable separation was observed along the three LDs, of spectral clusters belonging to the nine different times of development. Discriminant function 1 (LD1) was effective at separating developmental stages, although clear separation of DS02 from DS03, DS05 from DS06, and DS07 from DS08 was not observed (Fig. 3a). This indicates that spectral features of these stages show little to no differences with respect to the other developmental classes (DS01, DS04, and DS09). While DS02/DS03, DS05/DS06, and DS07/DS08 formed distinct clusters with no clear separation, these pairs were very distinct from one another effectively forming six distinguishable groups along LD1 (Fig. 3a). In contrast, discriminant LD2 showed a definitive separation of DS02 and DS03 but not of adjacent DS05/DS06 or DS07/DS08 (Fig. 3b). Separation of DS05 from DS06 was achieved along LD3 as opposed to no observable separation between DS07 and DS08 (Fig. 3c). Based on spectral data, it appears that DS07 and DS08 were most closely related as indicated by multivariate PCA-LDA of the first three LDs shown in entirety in Fig. 3. This is likely due to minimal changes occurring in the last few days of tomato fruit maturation, compared to changes occurring well before the mature green stage.

**Fig. 4:**
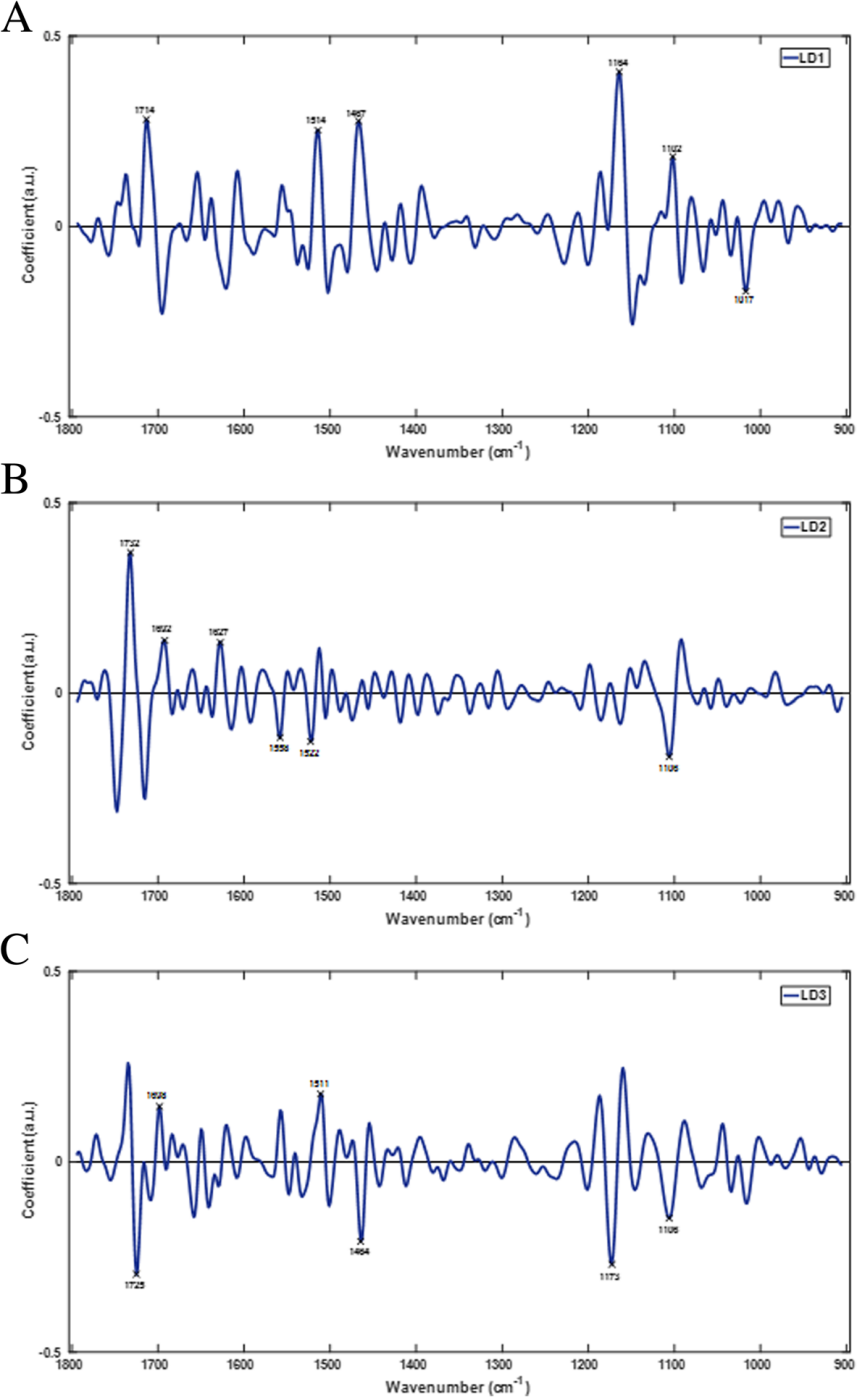
PCA-LDA loadings from the first three LDs; LD1 (**a**), LD2 (**b**), and LD3 (**c**) showing the top six discriminating wavenumbers responsible for group clustering of LD scores from developing tomato fruit (DS01-DS09)

In order to explore further the group clustering observed in the 3-dimensional discriminant space, PCA-LDA loadings were extracted for each of the three LDs to determine the specific spectral alterations associated with the tomato fruit developmental process. This provides a summary of the main biochemical changes occurring during tomato fruit development from DS01-DS09 between 4 and 36 dpa. Figure 4 shows PCA-LDA loadings (LD1-LD3) representing the main qualitative wavenumbers discriminating developmental stages of tomato fruit. The top six wavenumber biomarkers were selected from each loading to qualitatively characterize the biochemical compounds showing the greatest changes. Biomarkers extracted via PCA-LDA loadings provide potential biochemical and molecular markers for monitoring fruit development. Table 1 shows the top six discriminating wavenumbers for each of LD1–3 representing the main biochemical functional groups and associated compounds accompanying the developmental process in this cultivar. Specific changes were observed in the wavenumber regions 1732–1714, 1698–1627, 1558–1511, 1467–1464, 1173–1102, and 1017 cm^− 1^.

**Table 1:** Top six discriminating wavenumbers, corresponding vibrational modes, and biochemical assignments for the first three LDs as indicated by individual loadings of tomato development (Wavenumber references: [17, 23, 33, 35–37])

PCA-LDA Loadings	Wavenumber (cm^− 1^)	Vibrational Mode	Biochemical Assignment
LD1	1714	ν(C=O· · ·H) ester ν(C=O)	Cutin Phenolic compounds, pectin
	1514	ν(C-C) aromatic Amide II, ν(C=N), ν(C=C)	Phenolic compounds Proteins Lignin
	1467	δ(CH_2_) scissoring	Cutin, glycerolipids, wax hydrocarbons
	1164	ν_a_(C-O-C) ester ν(C-OH), ν(C-O-C)	Cutin Polysaccharide, cellulose
	1102	ν_s_(C-O-C) ester ν(C-O) ν_a_(PO_2_)	Cutin Pectin, cellulose, carbohydrates Phosphate
	1017	ν(C-O), ν(C-C) ν(C-OH)	Pectin, cellulose Pectin
LD2	1732	ν(C=O) ester ν(C=O)	Cutin, Lignin, wax, suberin-like aliphatic compounds
	1692	ν(C=O· · ·H weak) ν(C=O· · ·H strong)	Cutin Cutin
	1627	ν(C=C) phenolic acid ν(C=O) Amide I	Phenolic compounds De-esterified pectin Proteins
	1558	ν(C=C) phenolic acid	Phenolic compounds Proteins
	1522	ν(C-C) aromatic Amide II, ν(C=N), ν(C=C)	Phenolic compounds Proteins Lignin
	1106	ν_s_(C-O-C) ester ν(C-O) ν_a_(PO_2_)	Cutin Cellulose, pectin, carbohydrates Phosphate
LD3	1725	ν(C=O) ester	Cutin, pectin, phenolic compounds
	1698	ν(C=O· · ·H strong) ν(C=O· · ·H weak)	Cutin Cutin
	1511	ν(C-C) aromatic	Phenolic compounds Lignin
	1464	δ(CH_2_) scissoring	Cutin, glycerolipids, wax hydrocarbons
	1173	ν_a_(C-O-C) ester	Cutin
	1106	ν_s_(C-O-C) ester ν(C-O)	Cutin Pectin, cellulose, carbohydrates

### Spectral analysis of tomato fruit ripening

**Fig. 5:**
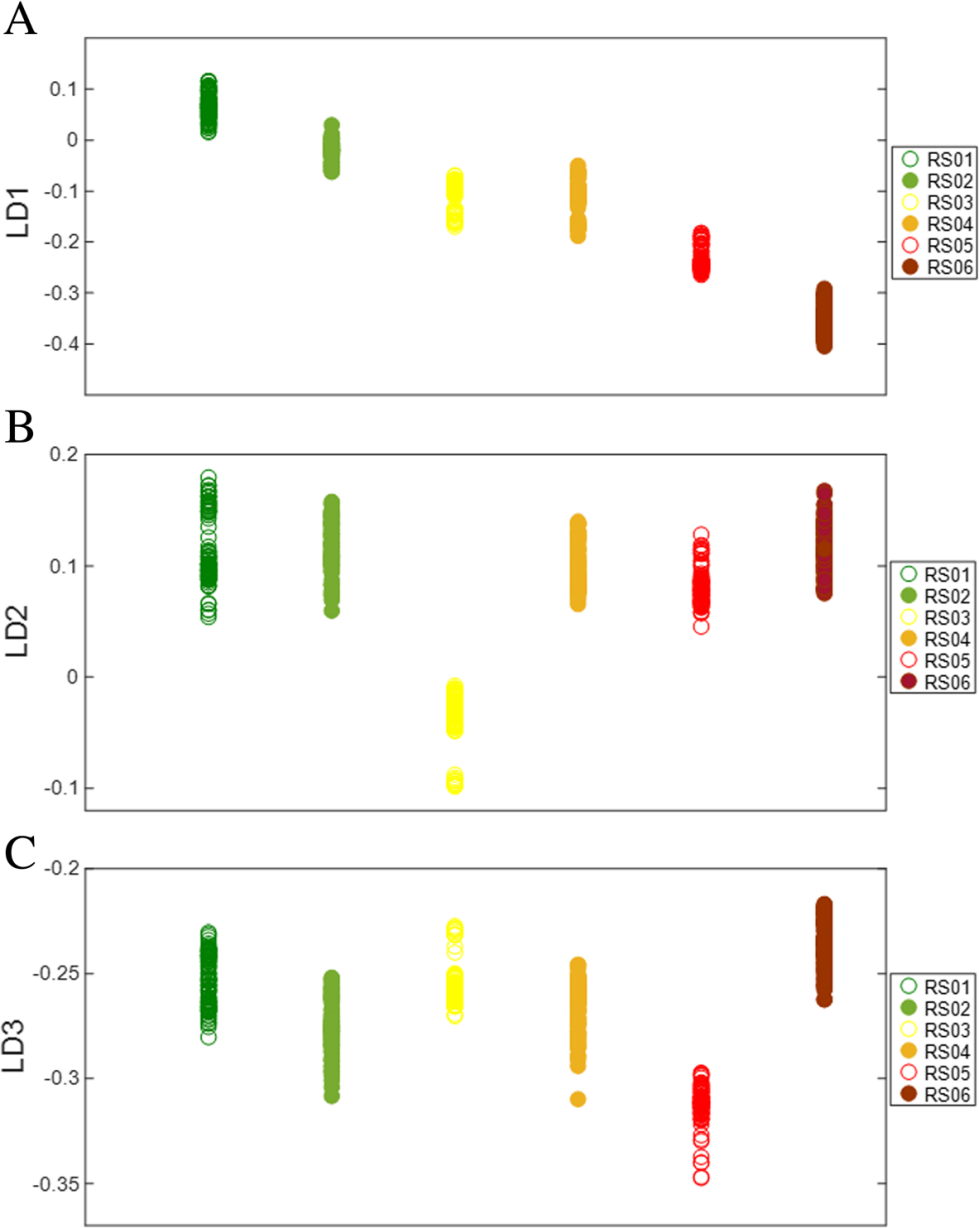
PCA-LDA 1-dimensional scores plots of tomato fruit ripening stages (RS01-RS06) along LD1 (**a**), LD2 (**b**), and LD3 (**c**)

Similar to multivariate analysis of developmental stage, ripening stages of tomato were also effectively distinguished along LD1, LD2, and LD3 (Fig. 5). These LDs were variably effective at separating the six distinct ripening stages. Most significant class separation was observed along LD1, with the six ripening stages showing clear separation, except RS03 and RS04, which showed no separation and were thus the most similar among these groups (Fig. 5a). Although LD1 was unable to separate RS03 from RS04, LD2 was highly effective at distinguishing RS03 from RS04 and all other ripening stages (Fig. 5b). Variable group clustering was seen along LD3, where RS05 was most clearly separated from other groups (Fig. 5c). As with tomato fruit development, ripening stages showed unique class clustering along the different LDs, indicating spectral features unique to each class. This raises the intriguing possibility that different LDs may be used in a targeted way to identify ripening stages in addition to different developmental stages based on this methodology.

**Fig. 6:**
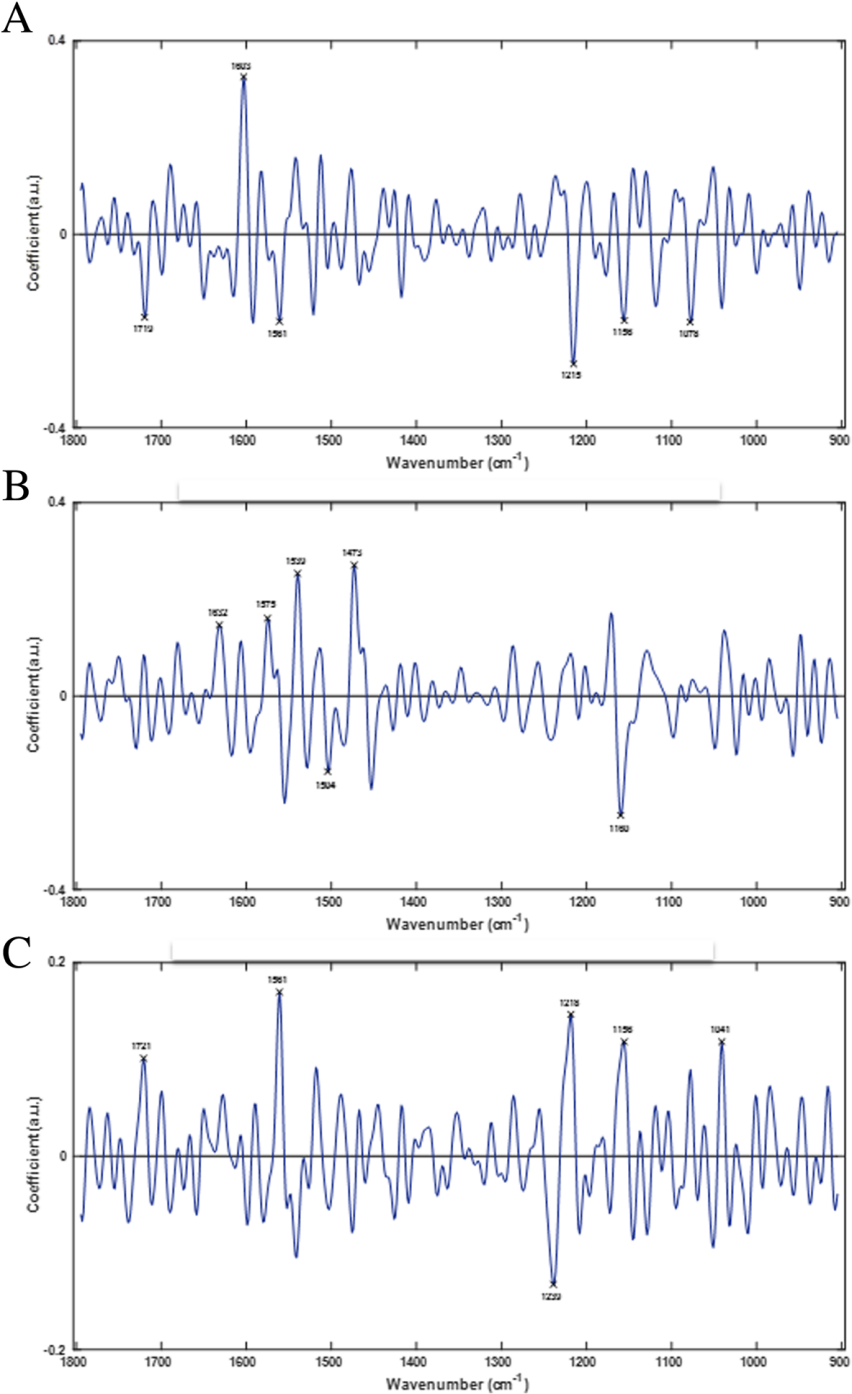
PCA-LDA loadings from the first three LDs; LD1 (**a**), LD2 (**b**), and LD3 (**c**) showing the top six discriminating wavenumbers responsible for group clustering of LD scores from ripening tomato fruit (RS01-RS06)

Spectral biomarkers for ripening extracted through PCA-LDA loadings, identified similar cuticle and cell wall components to those identified during development (Fig. 6); cutin, phenolic compounds, lipids, and waxes were identified at wavenumbers 1721, 1719, 1632, 1603 1561, 1473, 1160, and 1156 cm^− 1^ whilst lignin-like compounds, cellulose, pectin, and other polysaccharides were identified at wavenumbers 1719, 1603, 1539, 1504, 1239, 1160, 1156, 1078, and 1041 cm^− 1^ (Table 2). In addition, several unique spectral biomarkers related to proteins and indicative of ripening were identified, including the prominent Amide I, II, and III regions. Proteins are prominent components of the cell wall, and to lesser extent plant cuticles, suggesting that these proteins may be ripening-dependent based on the multiple protein vibrations seen over the fingerprint spectrum. Protein vibrational modes were seen specifically at wavenumbers 1632, 1575, 1539, 1239, and 1218 cm^− 1^ (Table 2). These changes may be associated with the softening of the fruit skin and the depolymerisation of pectin and other natural polymers during ripening [38] resulting in alterations to both the accessibility and abundance of proteins embedded in the cell wall-cuticle complex. The (C-H) vibration at 1504 cm^− 1^ is potentially linked to an increase in the carotenoid content, and specifically lycopene, during ripening. The ripening-specific biomarker at 1078 cm^− 1^ has previously associated with xyloglucan and is also likely to be associated with xyloglucosyltransferase/endohydrolase (XTH) activity in the epidermis, which has an active role in fruit softening in tomato [39]. Interestingly, wavenumber 1041 cm^− 1^ was associated with arabinogalactan. Arabinogalactan-glycoproteins at the plant cell surface have been implicated in plant growth and development and may integrate changes occurring in the cell wall and cuticle layers during ripening [40, 41]. The detection of protein vibrations, which may signify increased protein abundance during tomato ripening, reinforces the identity of arabinogalactan as part of glycoproteins, and provides a link between xyloglucan and XTH enzyme activity during the ripening program.

**Table 2:** Top six discriminating wavenumbers, corresponding vibrational modes, and biochemical assignments for the first three LDs as indicated by individual loadings of tomato fruit ripening. (Wavenumber references: [17, 23, 33, 35–37])

PCA-LDA Loadings	Wavenumber (cm^− 1^)	Vibrational Mode	Biochemical Assignment
LD1	1719	ν(C=O· · ·H) ester ν(C=O)	Cutin Pectin, lipid, polysaccharides, phenolic compounds
	1603	ν(C-C) aromatic ν(COO), ν(C=C)	Phenolic compounds Pectin, lignin
	1561	ν(C-C) aromatic	Phenolic compounds
	1215	ν_a_PO_2_ Amide III	Phosphate Proteins
	1156	ν(C-OH) ν_a_(C-O-C) ester	Cellulose, polysaccharide Cutin, pectin
	1078	ν(C-O), ν(C-C) ν(C-OH) ν_s_PO_2_	Xyloglucan Oligosaccharide Phosphate
LD2	1632	ν(C=C) phenolic acid ν_s_(C-C) ring Amide I	Phenolic compounds Cellulose Proteins
	1575	ν(C=N) ν(C-C) phenyl group	Proteins Phenolic compounds
	1539	Amide II ν(C=N)	Proteins Lignin
	1504	ν(C=C) ν(C-H) Amide II	Lignin Carotenoid Protein
	1473	δ(CH_2_) scissoring	Glycerolipids, wax hydrocarbons
	1160	ν_a_(C-O-C) ester ν(C-OH), ν(C-O-C)	Cutin Cellulose, polysaccharide
LD3	1721	ν(C=O) ester ν(C=O)	Cutin, pectin Phenolic compounds, lipids, polysaccharides
	1561	ν(C-C) aromatic	Phenolic compounds
	1239	Amide III ν(C-O)	Proteins Pectin
			Cellulose / hemicellulose
		ν_a_PO_2_	Phosphate
	1218	Amide III ν_a_PO_2_	Proteins Phosphate
	1156	ν_a_(C-O-C), ester ν(C-OH), ν(C-O-C)	Cutin Polysaccharide, cellulose
	1041	ν(C-O), ν(C-C) ν(O-CH_2_)	Cellulose Arabinogalactan

### Autonomous determination of tomato fruit Development and ripening stages

Classification using PCA-LDA was satisfactory for the tentative assignment of spectral biomarkers but SVM was required for more effective classification of developmental and ripening stages. Autonomous sorting of tomato fruit based on their spectral characteristics is an exciting possibility. To test the feasibility of using classification performance based on fingerprint spectra, both PCA-LDA and SVM were applied (Additional file 1: Table S1, Additional file 2: Table S2, Additional file 3: Table S3; Additional file 4: Figure S1 and Additional file 5: Figure S2). While the classification performance of PCA-LDA was satisfactory (Additional file 1: Table S1), this approach was used primarily for biomarker extraction. To improve classification performance from that achieved by PCA-LDA, SVM was applied. While SVM was superior for sheer classification purposes, SVM is a non-linear method and therefore does not provide biochemical information. Table 3 shows the results for SVM based autonomous classification of developmental stages based on ATR-FTIR fingerprint spectra. High accuracy was observed for all developmental grades of tomato, with minimal misclassification only between directly related late stages of development (DS08 and DS09) between ~ 34–36 dpa as shown in the confusion matrix for development (Table 3). Sensitivity and specificity rates for development were correspondingly high (Additional file 2: Table S2 and Additional file 4: Figure S1). These results indicate that changes in the epidermal surfaces are sufficient to determine with exceptionally high sensitivity and specificity, the developmental stage of whole tomato fruit non-destructively with compact equipment. Further, tomato development can be distinguished, in this case within ±4 days of the next developmental stage.

**Table 3:** Confusion matrix showing predictive performance calculated for the SVM chemo-metric model intended to differentiate tomato fruit developmental stages from their ATR-FTIR spectral data

	DS01	DS02	DS03	DS04	DS05	DS06	DS07	DS08	DS09
DS01	100%	0%	0%	0%	0%	0%	0%	0%	0%
DS02	0%	100%	0%	0%	0%	0%	0%	0%	0%
DS03	0%	0%	100%	0%	0%	0%	0%	0%	0%
DS04	0%	0%	0%	100%	0%	0%	0%	0%	0%
DS05	0%	0%	0%	0%	100%	0%	0%	0%	0%
DS06	0%	0%	0%	0%	0%	100%	0%	0%	0%
DS07	0%	0%	0%	0%	0%	0%	100%	0%	0%
DS08	0%	0%	0%	0%	0%	0%	0%	99%	1%
DS09	0%	0%	0%	0%	0%	0%	0%	0%	100%

Tomato fruit are harvested at different developmental stages depending on their end use to ensure the desired qualities unique to each developmental. There are at least 4 maturity grades of tomato harvested between 4 to 36 dpa (M1-M4) providing fruit of different quality at maturity [42, 43]. Currently, the horticultural industry typically relies on subjective visual and/or destructive determination of maturity and ripening stages for tomato grading [44]. Therefore, the development of objective and non-destructively approaches to determine fruit maturity and ripening stage, while gaining valuable biochemical information, would be beneficial to the industry. Here we provide evidence that ATR-FTIR combined with chemometric modelling can classify many distinct developmental stages, in this case nine, without destructive measurement but high selectivity and specificity (Table 3 and Additional file 2: Table S2). These results exceed or are at least on par with other spectroscopic approaches currently used to assess fruit maturity and quality parameters [29, 30, 32].

Six horticultural ripening grades are typically distinguished based on colour schemes; mature green, breaker, turning, pink, light red, and red [43]. Spectral data combined with chemometrics was also effective at identifying the six distinct ripening grades of tomato. As with developmental groups, spectra of the six ripening grades were subjected to SVM analysis (Additional file 3: Table S3 and Additional file 5: Figure S2). Table 4 shows that the six ripening grades were distinguished with almost between 99 and 100% accuracy, the only exception being between the adjacent ripening grades ‘turning’ and ‘pink’.

**Table 4:** Confusion matrix showing predictive performance calculated for the SVM chemometric model intended to differentiate tomato fruit ripening stages from their ATR-FTIR spectral data

	Mat. Green	Breaker	Turning	Pink	Light Red	Red Ripe
Mat. Green	100%	0%	0%	0%	0%	0%
Breaker	0%	100%	0%	0%	0%	0%
Turning	0%	0%	99%	1%	0%	0%
Pink	0%	0%	0%	100%	0%	0%
Light Red	0%	0%	0%	0%	100%	0%
Red Ripe	0%	0%	0%	0%	0%	100%

## Discussion

### Spectral characteristics of tomato fruit Development

Discriminant analysis reveals class-dependent clustering of spectral groups and allows the extraction of qualitative biomarkers. ATR-FTIR probes the first few microns of the sample, which in plants constitutes the external epidermal layers, and therefore provides an overview of the biochemical changes at the plant-environment interface during fruit development. The cutinized cell wall, which forms a biochemically complex heterogeneous matrix as part of the outer epidermis [12], is composed of various soluble waxes embedded in the main polymer Cutin (~ 40–80%), along with a small phenolic fraction (~ 1–5%) [13, 45]. The underlying cell wall consists mainly of cellulose, pectin, various polysaccharides, and proteins [12]. During tomato fruit development, the cuticle and cell wall undergo structural and compositional changes that are distinct to the stage of development, including the transition from cell division to cell expansion, cuticle biogenesis, and changes in cell wall thickness [11]. Consequently, the relative contributions of the cell wall and cuticle to the epidermal plant surface varies markedly during fruit development due to rapid cell division (2 to 35–40 dpa) and the subsequent cell expansion [46]. These surface layers therefore present unique in vivo molecular targets for distinguishing between developmental stages using ATR-FTIR spectroscopy.

Multi-component analysis over the fingerprint spectrum (1800–900 cm^− 1^) showed that the alterations in these spectral regions were strongly associated with both prominent cuticle and cell wall components including their main constituents. Spectral biomarkers strongly associated with cutin, waxes, and phenolic compounds were all detected (Table 1) and are consistent with changes in the cuticle during development 4–36 dpa [45, 47, 48]. Cutin was identified at wavenumbers 1732, 1725, 1714, 1698, 1692, 1467, 1464, 1173, 1164, 1106, and 1102 cm^− 1^. Waxes, including glycerolipids and suberin-like compounds, were identified at wavenumbers 1732, 1467, and 1464 cm^− 1^. Primary phenolic compounds were identified at wavenumbers 1627, 1558, 1522, 1514, and 1511 cm^− 1^. Other spectral alterations observed originated from cell wall components as part of the cutinized cell wall structure. Spectral biomarkers associated with the cell wall identified cellulose, pectin, and various other carbohydrate moieties. Cellulose and pectin were related to wavenumbers at 1725 1714, 1106, 1102, and 1017 cm^− 1^ respectively. Other carbohydrates, including some lignin like compounds, showed overlap with pectin, cellulose, and other moieties at wavenumbers 1522, 1514, 1511, 1164, 1106, and 1102 cm^− 1^. These results show clearly the power of multivariate analysis of fingerprint spectra to provide information about the biochemical changes occurring in the cuticle and cell wall during tomato fruit development. However, further work is needed to decipher the exact role of these compounds in the context of their IR absorptive properties. Importantly, the developmental time frame between 4 and 36 dpa contains at least four horticultural grades used as industry standards; the maturity grades M1-M4 correspond approximately to DS06-DS09 [42–44]. Therefore, the preliminary characterization, qualitative analysis presented here shows the potential for distinguishing developmental stages according to horticultural grades, for example the mentioned M1-M4 grades, based on their epidermal surface properties through the detection of multitude spectral biomarkers related to tomato fruit development. Nevertheless, further investigations are still required to determine the structure-function relationships in tissues at different developmental stages and for horticultural applications such as maturity grading.

### Spectral characteristics of tomato fruit ripening

Ripening, although part of the natural development of tomato fruit, is often seen as a separate developmental stage due to it separate genetic regulation and distinct colour changes. Significant shifts in gene expression, and transition in ethylene biosynthesis result in modifications of epidermal surfaces of tomato fruit during ripening, which influence post-harvest qualities [46, 48, 49]. Changes in the epidermal layers of tomato fruit thereby differ significantly from the developmental phase and throughout the ripening period. Figure 4 shows that during ripening, tomato fruit exhibit both unique and common spectral features from those observed for development that separate into distinct spectral clusters corresponding to the six ripening stages: mature green, breaker, turning, pink, light red, and red ripe (RS01-RS06, see Fig. 1).

Distinct biophysical and associated biochemical changes occur in the cuticle and cell wall during tomato ripening approx. 35–55 dpa [11, 50]. The observed spectral changes in the epidermal layers are therefore likely to be associated with events including cuticle rearrangement, cell wall disassembly, carotenoid accumulation, and the underpinning changes in genetic and metabolic regulation [12, 49]. Consequently, this exploratory analysis clearly shows that biospectroscopy can provide an abundance of chemical information that can contribute to understanding of the changes that occur in the epidermal layers during development and ripening. Importantly, the ability to analyse intact fruit will enable baseline characterizations of the development and ripening of healthy fruit, offering the intriguing possibility of using deviations from the baseline as indicators of abnormal development, stress, or disease. For horticultural applications, ripening is particularly interesting as the late red ripe stage are key stages for consumer consumption but are also stages at which fruit become increasingly susceptible to events such as fruit cracking and pathogen infection, which are linked directly to epidermal structure and fruit integrity [10, 51].

### Common spectral characteristics of tomato fruit Development and ripening

Tomato fruit development and ripening show common spectral features relating primarily to the cuticle and cell wall components. Discriminant wavenumbers common to both fruit development and ripening include the general regions: 1725–1714, 1632–1627, 1561–1558, 1514–1511, and 1473–1464 cm^− 1^ (Tables 2 and 3). Specifically, these spectral biomarkers include wavenumbers at 1725, 1721, 1719, and 1714 cm^− 1^, which are strong absorbance contributions of ν(C=O) ester and medium absorbance contribution from ν(C=O· · ·H) ester of Cutin. Wavenumbers at 1632 and 1627 cm^− 1^ were medium and strong absorbance of ν(C=C) respectively, which were indicative of phenolic compounds. Further weak ν(C-C) absorbance of phenolic compounds was seen at 1561 and1558 cm^− 1^, while regions including 1514 and 1511 cm^− 1^ related to ν(C-C)~(C=C) conjugated aromatic entities of phenolic compounds. In the traditional context of IR spectroscopy, these absorbance designations strong, medium, and weak, refer to the highest, intermediate, and smallest peak amplitudes respectively, relative to one another in the spectrum [52]; these reflect both the IR activity of functional groups and their abundance in the sample. Common development and ripening spectral biomarkers identified here in intact tomato fruit have also been observed in isolated cuticles of both immature green and red ripe tomato fruit [33]. Similarities seen at 1473, 1467, and 1464 cm^− 1^ were interpreted as indicating δ(CH_2_) scissoring of Cutin and waxes present in mature and immature fruit cuticles [33, 53]. Cutin is one of the outermost and abundant compounds in the cuticle and therefore the spectral changes observed in both the intact fruit and isolated cuticles are consistent with the changes in cuticular compounds, and especially Cutin, during development and ripening. ATR-FTIR spectral analysis as a surface technique identifies common biomarkers in the surface layers of fruit across the total developmental program from ~ 4–55 dpa, which are associated mainly with changes in the cuticle. However, novel regions in the fingerprint spectrum may provide insights into the exact role of the many cuticle functions in fruit development and ripening [10].

### Identifying tomato fruit Development and ripening stages

The ability to distinguish nine distinct developmental stages and six common horticultural ripening grades of tomato fruit autonomously and non-destructively represents an important advance enabling expert growers or industrial food production/supply chains to grade fruit quality more effectively. Sensor-based horticultural systems will rely on multiple inputs from various sensors. For this reason, it is important to explore and employ different sensors and see these as being complementary rather than competitive. Various studies have shown that tomato maturity grading and assessing quality parameters can be achieved using spectroscopies that employ different wavelengths and ranges of the electromagnetic spectrum between UV, visible and IR light. To contribute to the expansion of MIR sensors, it was shown that the present level of accuracy was achieved using a compact spectrometer, relatively small data set compared to the number of samples available for testing in a horticultural setting, where typically classification accuracy increases with larger datasets. Although external validation is necessary to solidify these results, this study provides a clear indication of the potential, specifically ATR-FTIR for automatic classification of various horticultural products including tomato.

## Conclusions

Biospectroscopy is a powerful analytical tool and potential sensor technology for linking fundamental plant biology and applied crop sciences as part of developing precision horticulture systems. The development of surface techniques including MIR spectroscopy that are applicable to both homogenous and for heterogeneous substances has opened the door for analysis of intact tissues and non-destructive measurements in vivo. However, to date the degree to which MIR spectroscopy has been used to study intact plants has been limited, as has the evaluation of portable equipment that may be readily retooled for use in horticultural applications [25]. The ATR-FTIR sampling mode, probes the main groups of biochemical compounds within tomato fruit epidermal surface layers, such as cutin, wax, and phenolic fractions of the cuticle, as well as cellulose, pectin, carbohydrates, and lignin-like compounds as primary cell wall constituents (Tables 1 and 2), and is thus ideal for the study of plant epidermis as it relates to horticultural parameters. Biospectroscopy based multi-compound analysis, within plant organs in vivo, offers an alternative methodology to conventional ways of studying cuticle and cell wall structure during development or in response to industrial processing [33, 36]. In this regard, MIR biospectroscopy will prove useful for deciphering the molecular details of changing epidermal structures during tomato fruit development and ripening. This is critical because the detailed mechanisms behind cuticle formation are debated, and little is known about the relationship between cuticle structure and postharvest characteristics in whole tomato fruit [10, 13].

As a method for in vivo analysis, as demonstrated here on delicate tomato fruit, ATR-FTIR spectroscopy can measure large groups of compounds in epidermal structures of whole tomato fruit. Exploratory discriminant analysis (PCA-LDA) associated these groups of compounds with specific biomarkers of tomato fruit development and ripening identifying both common and unique spectral features reflecting the distinct changes occurring during tomato fruit development and ripening. The various compounds reflected by the fingerprint spectra can be tentatively assigned to components from epidermal surface layers including the cuticle and cell wall. As part of the intact cutinized cell wall, compounds including Cutin, waxes, phenolics, cellulose, pectin, and lignin were present, which showed major alterations although qualitative interpretation of spectral biomarkers remains challenging due to limitations in our knowledge of how the cell wall-cuticle complex changes during fruit development [11, 13]. Nevertheless, epidermal layers play important roles in the quality of fruit, as well as in the determination of horticultural grades at various points of tomato fruit development [10]. Automatic grading of the defined tomato fruit groups was evaluated using the SVM classification model indicating that development and ripening can be distinguished at a minimum of 15 separate stages (9 for development and 6 for ripening). Importantly, all analyses were entirely non-destructive and were performed using a compact portable ATR-FTIR spectrometer suggesting the potential for field-based analysis.

Most elements needed to transition this approach from a lab-based analytical method to an applied sensor technology for routine monitoring are already available including portable spectrometers, fast data analysis tools, and the minimal to no sample preparation required for most crop plants making this a realistic possibility. To realise this potential, application of biospectroscopy to additional model plant systems is needed alongside the evaluation of new portable equipment, similar to that recently developed for Raman spectroscopy [25]. With these advances, rapid analysis with optical sensors such as MIR spectroscopy will further permit the automatic characterization of healthy fruit development, and enabling abnormalities related to damage or disease to be reliably identified. In addition, further development of biospectroscopy in the plant and crop sciences will contribute to a better biological and biochemical understanding of plant surface layers, and how these affect the traits of plant organs such as fruit; thereby, contributing to both molecular plant biology and industrial horticulture for better crop production.

## Methods

### Plant growth conditions

Individual tomato plants, *Solanum lycopersicum* cv. Moneymaker, were grown from commercial seed (Thompson and Morgan Seeds, UK) in 10 L pots containing Levington’s M3 growth medium (Levington Horticulture Ltd., Ipswich, UK) to anthesis (approx. 60 days). Plants were grown in a heated glasshouse (25 ± 5 °C) with an 18/6 h day/night cycle (minimum illumination 500 μmol m^− 2^ s^− 1^ at the plant canopy from 600 W metal-halide lamps) and 50 ± 10% humidity. Tomorite fertilizer (Levington Horticulture Ltd., Ipswich, UK) was applied from anthesis, at every other watering according to the manufacture’s instructions. Criteria for development and ripening stages was dpa, where the initial class was measured at 4 dpa and subsequent classes were separated by 4 days of growth for both the development and ripening series respectively. Tomato fruit parameters used in the selection process were recorded for development and ripening sets corresponding to those shown in Fig. 1 and are found in Additional file 6: Table S4 and Additional file 7: Table S5.

### ATR-FTIR spectroscopy

Tomato fruit were picked from plants, washed with deionized water, dried and immediately measured using ATR-FTIR spectroscopy. Vibrational spectra were acquired from intact tomato fruit at 9 developmental stages (DS01-DS09) and 6 ripening stages (RS01-RS06). Whole tomato fruit were placed on the sample stage for analysis, with no more than 0.1 kg of applied pressure to ensure adequate sample contact. Five points from each fruit were measured around the circumference; two spectra were taken at each contact point for a total of 10 measurements per fruit. Ten fruits were analysed, for a total of 100 spectra for each developmental and ripening stage making a total of 900 spectra for the development dataset (9 classes) and 600 spectra for the ripening dataset (6 classes). Spectra were acquired using a compact portable Bruker Alpha-P infrared spectrometer with platinum ATR attachment (Bruker Optics, Coventry, UK), over the range 4000–400 cm^− 1^ with a spectral resolution of 8 cm^− 1^, 32 co-additions and a mirror velocity of 7 kHz. Background spectra were taken prior to sample measurement to account for ambient atmospheric conditions. The diamond ATR crystal defined a spatial resolution (sampling area) of 1 mm^2^ and was cleaned between measurements with isopropyl-alcohol ATR cleaning wipes (Bruker Optics, Coventry, UK).

### Computational analysis

Raw spectra truncated to the spectral fingerprint region (1800–900 cm^− 1^) were pre-processed using the Savitzky-Golay filter and second order differentiation, followed by vector normalization to account for differences in sample thickness and ATR diamond contact pressure. PCA-LDA analysis was used for exploratory data analysis and biomarker extraction. PCA-LDA analysis was performed using the open source IRootlab toolbox (https://github.com/trevisanj/irootlab) specialized for analysis of IR spectra [54], in conjunction with Matlab 2016a (The Maths Works, MA, USA). Principal component analysis reduces the dataset down to factors that account for spectral variance; PCA was optimized using IRootlab to ensure the inclusion of the primary dataset variance within the first 10 PCs. The first 10 PCs accounted for more than 97 and 95% of variance in the development and ripening datasets, respectively (Additional file 8: Table S6). These served as input variables for LDA forming the composite technique PCA-LDA [21]. Exploratory analysis by way of cluster separation along the three main linear discriminants (LD1, LD2, and LD3) was explored, to determine whether specific clustering of spectral groups, belonging to developmental and ripening stages, could be observed. PCA-LDA scores were cross validated 10 k-folds. For a qualitative characterization of the main spectral alterations, PCA-LDA loadings in combination with a peak-pick algorithm (20 cm^− 1^ minimum separation) was used to tabulate the top six most prominent vibrational mode alterations, and their corresponding chemical assignments, which were used as tentative biomarkers for development and ripening [22].

Testing of classification accuracy of DS01-DS09 and separately RS01-RS06 stages with SVM was conducted using the PLS toolbox version 7.9 (Eigenvector Research, Inc., WA, USA); in conjunction with Matlab 2016a. Classification of developmental and ripening stages was performed using an SVM classifier. The SVM classifier was constructed using 90% of data for training and 10% for internal validation. The same data used for PCA-LDA, pre-processed fingerprint spectra, were used as input for SVM. This model was developed to improve on the classification performance of PCA-LDA (Additional file 1: Table S1, Additional file 2: Table S2, Additional file 3: Table S3, Additional file 4: Figure S1 and Additional file 5: Figure S2). SVM was cross-validated using 10 k-folds.

## Additional files

Additional file 1: **Table S1.** Predictive performance presented as sensitivity and specificity rates calculated for the PCA-LDA chemometric model intended to differentiate tomato fruit developmental and ripening stages from their ATR-FTIR spectral data. (DOCX 13 kb)

Additional file 2: **Table S2.** Predictive performance presented as sensitivity and specificity rates calculated for the SVM chemometric model intended to differentiate tomato fruit developmental stages from their ATR-FTIR spectral data. (DOCX 12 kb)

Additional file 3: **Table S3.** Predictive performance presented as sensitivity and specificity rates calculated for the SVM chemo-metric model intended to differentiate tomato fruit ripening stages from their ATR-FTIR spectral data. (DOCX 12 kb)

Additional file 4: **Figure S1.** Class predictive performance SVM for development classes. (PPTX 68 kb)

Additional file 5: **Figure S2.** Class predictive performance SVM for ripening classes. (PPTX 70 kb)

Additional file 6: **Table S4.** Development stages of tomato fruit *S. lycopersicum* (cv. Moneymaker), corresponding spectral classes, and their AMS (USDA) grade designation (Kader and Morris [44]; Sargent [43]; Maul et al. [42]). (DOCX 13 kb)

Additional file 7: **Table S5**. Ripening stages of tomato fruit *S. lycopersicum* (cv. Moneymaker), corresponding AMS (USDA) ripening and spectral class designation (Sargent [43]; Maul et al. [42]). Fruit used for ripening stages had an average diameter of 7.31 ± 0.24 cm. (DOCX 12 kb)

Additional file 8: **Table S6**. Percentage of variance for PCA-LDA models varying the number of PCs. (DOCX 17 kb)

## Data Availability

The datasets used and/or analysed during the current study are available from the corresponding author on reasonable request**.**

## Abbreviations

ATR: Attenuated Total Reflection
DS: Developmental Stage
FTIR: Fourier-transform Infrared
LDA: Linear Discriminant Analysis
MIR: Mid-infrared
PCA: Principal Component Analysis
RS: Ripening Stage
SVM: Support Vector Machine
