# Supplementary material for: Determination of developmental and ripening stages of whole tomato fruit using portable infrared spectroscopy and Chemometrics
Source: BMC Plant Biol. 2019 Jun 4;19:236. doi: 10.1186/s12870-019-1852-5 (PMC6549295; doi:10.1186/s12870-019-1852-5)
Supplement: Supplementary file 1 — Table S1. Predictive performance presented as sensitivity and specificity rates calculated for the PCA-LDA chemometric model intended to differentiate tomato fruit developmental and ripening stages from their ATR-FTIR spectral data. (DOCX 13 kb) [file 12870_2019_1852_MOESM1_ESM.docx]

**Additional File 1**

Table S1: Predictive performance presented as sensitivity and specificity rates calculated for the PCA-LDA chemometric model intended to differentiate tomato fruit developmental and ripening stages from their ATR-FTIR spectral data.

|  | **Grouped data** | | **Cross-validation** | |
| --- | --- | --- | --- | --- |
| **Developmental Stage (dpa)** | **Sensitivity** | **Specificity** | **Sensitivity** | **Specificity** |
| DS01 (04) | 100% | 100% | 100% | 100% |
| DS02 (08) | 63% | 99% | 63% | 99% |
| DS03 (12) | 92% | 95% | 92% | 95% |
| DS04 (16) | 85% | 100% | 84% | 100% |
| DS05 (20) | 79% | 99% | 79% | 99% |
| DS06 (24) | 90% | 98% | 90% | 98% |
| DS07 (28) | 69% | 98% | 69% | 98% |
| DS08 (32) | 86% | 93% | 86% | 93% |
| DS09 (36) | 97% | 100% | 97% | 100% |
| **Ripening Stage** | **Sensitivity** | **Specificity** | **Sensitivity** | **Specificity** |
| Mature Green | 100% | 100% | 100% | 100% |
| Breaker | 100% | 98% | 100% | 98% |
| Turning | 100% | 100% | 100% | 100% |
| Pink | 90% | 100% | 89% | 100% |
| Light Red | 100% | 100% | 100% | 100% |
| Red | 100% | 100% | 100% | 100% |
