# Supplementary material for: Determination of developmental and ripening stages of whole tomato fruit using portable infrared spectroscopy and Chemometrics
Source: BMC Plant Biol. 2019 Jun 4;19:236. doi: 10.1186/s12870-019-1852-5 (PMC6549295; doi:10.1186/s12870-019-1852-5)
Supplement: Supplementary file 2 — Table S2. Predictive performance presented as sensitivity and specificity rates calculated for the SVM chemometric model intended to differentiate tomato fruit developmental stages from their ATR-FTIR spectral data. (DOCX 12 kb) [file 12870_2019_1852_MOESM2_ESM.docx]

**Additional File 2**

Table S2: Predictive performance presented as sensitivity and specificity rates calculated for the SVM chemometric model intended to differentiate tomato fruit developmental stages from their ATR-FTIR spectral data.

| **Developmental Stage (dpa)** | **Sensitivity** | **Specificity** |
| --- | --- | --- |
| DS01 (04) | 100% | 100% |
| DS02 (08) | 100% | 100% |
| DS03 (12) | 100% | 100% |
| DS04 (16) | 100% | 100% |
| DS05 (20) | 100% | 100% |
| DS06 (24) | 100% | 100% |
| DS07 (28) | 100% | 100% |
| DS08 (32) | 100% | 99% |
| DS09 (36) | 99% | 100% |
