# Supplementary material for: Determination of developmental and ripening stages of whole tomato fruit using portable infrared spectroscopy and Chemometrics
Source: BMC Plant Biol. 2019 Jun 4;19:236. doi: 10.1186/s12870-019-1852-5 (PMC6549295; doi:10.1186/s12870-019-1852-5)
Supplement: Supplementary file 3 — Table S3. Predictive performance presented as sensitivity and specificity rates calculated for the SVM chemo-metric model intended to differentiate tomato fruit ripening stages from their ATR-FTIR spectral data. (DOCX 12 kb) [file 12870_2019_1852_MOESM3_ESM.docx]

**Additional File 3**

Table S3: Predictive performance presented as sensitivity and specificity rates calculated for the SVM chemo-metric model intended to differentiate tomato fruit ripening stages from their ATR-FTIR spectral data.

| **Ripening Stage** | **Sensitivity** | **Specificity** |
| --- | --- | --- |
| Mature Green | 100% | 100% |
| Breaker | 100% | 100% |
| Turning | 100% | 99% |
| Pink | 99% | 100% |
| Light Red | 100% | 100% |
| Red | 100% | 100% |
