# Supplementary material for: Determination of developmental and ripening stages of whole tomato fruit using portable infrared spectroscopy and Chemometrics
Source: BMC Plant Biol. 2019 Jun 4;19:236. doi: 10.1186/s12870-019-1852-5 (PMC6549295; doi:10.1186/s12870-019-1852-5)
Supplement: Supplementary file 4 — Figure S1. Class predictive performance SVM for development classes. (PPTX 68 kb) [file 12870_2019_1852_MOESM4_ESM.pptx]

## Slide 1
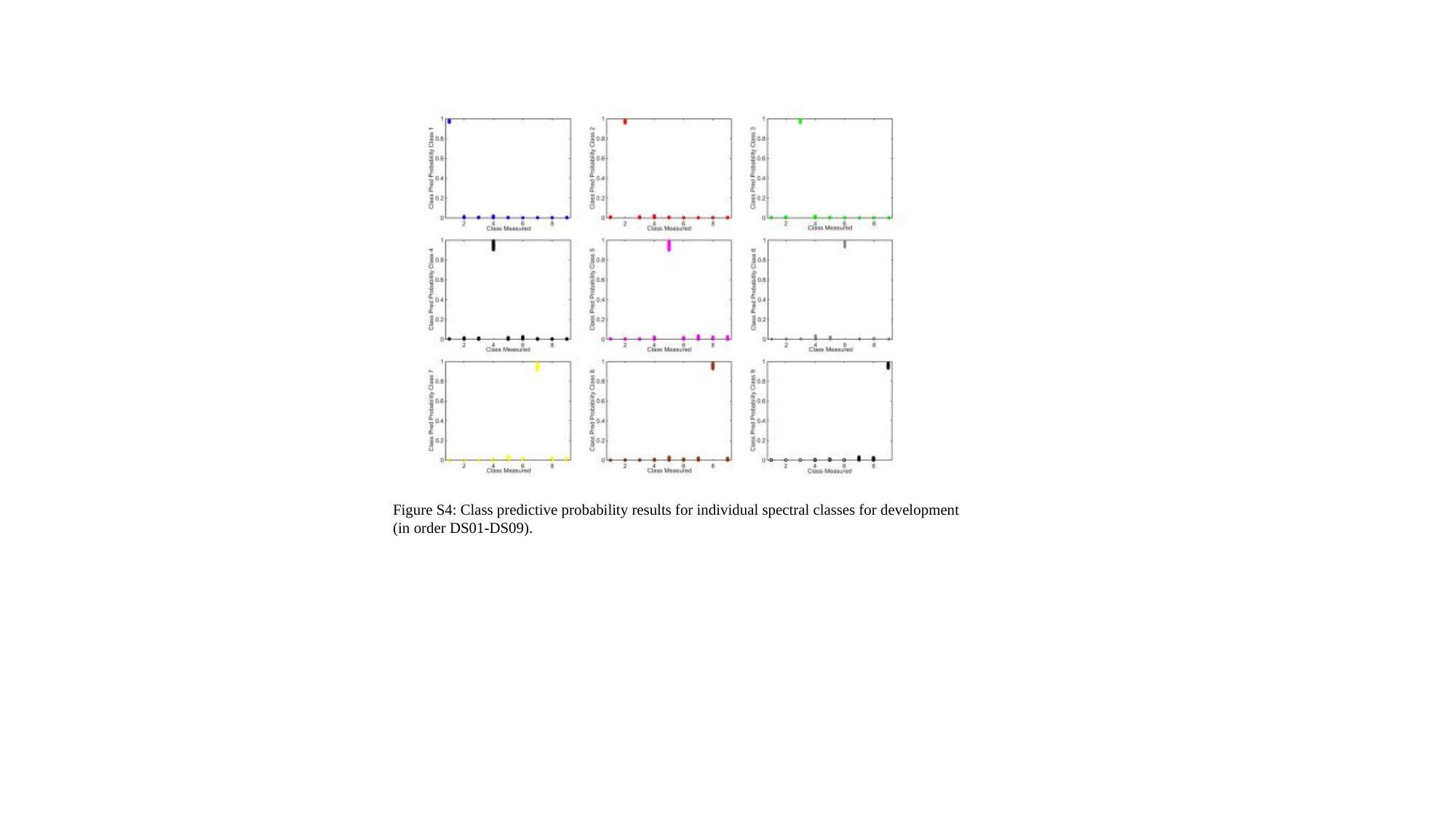

Figure S4: Class predictive probability results for individual spectral classes for development
(in order DS01-DS09).
