# Supplementary material for: Determination of developmental and ripening stages of whole tomato fruit using portable infrared spectroscopy and Chemometrics
Source: BMC Plant Biol. 2019 Jun 4;19:236. doi: 10.1186/s12870-019-1852-5 (PMC6549295; doi:10.1186/s12870-019-1852-5)
Supplement: Supplementary file 5 — Figure S2. Class predictive performance SVM for ripening classes. (PPTX 70 kb) [file 12870_2019_1852_MOESM5_ESM.pptx]

## Slide 1
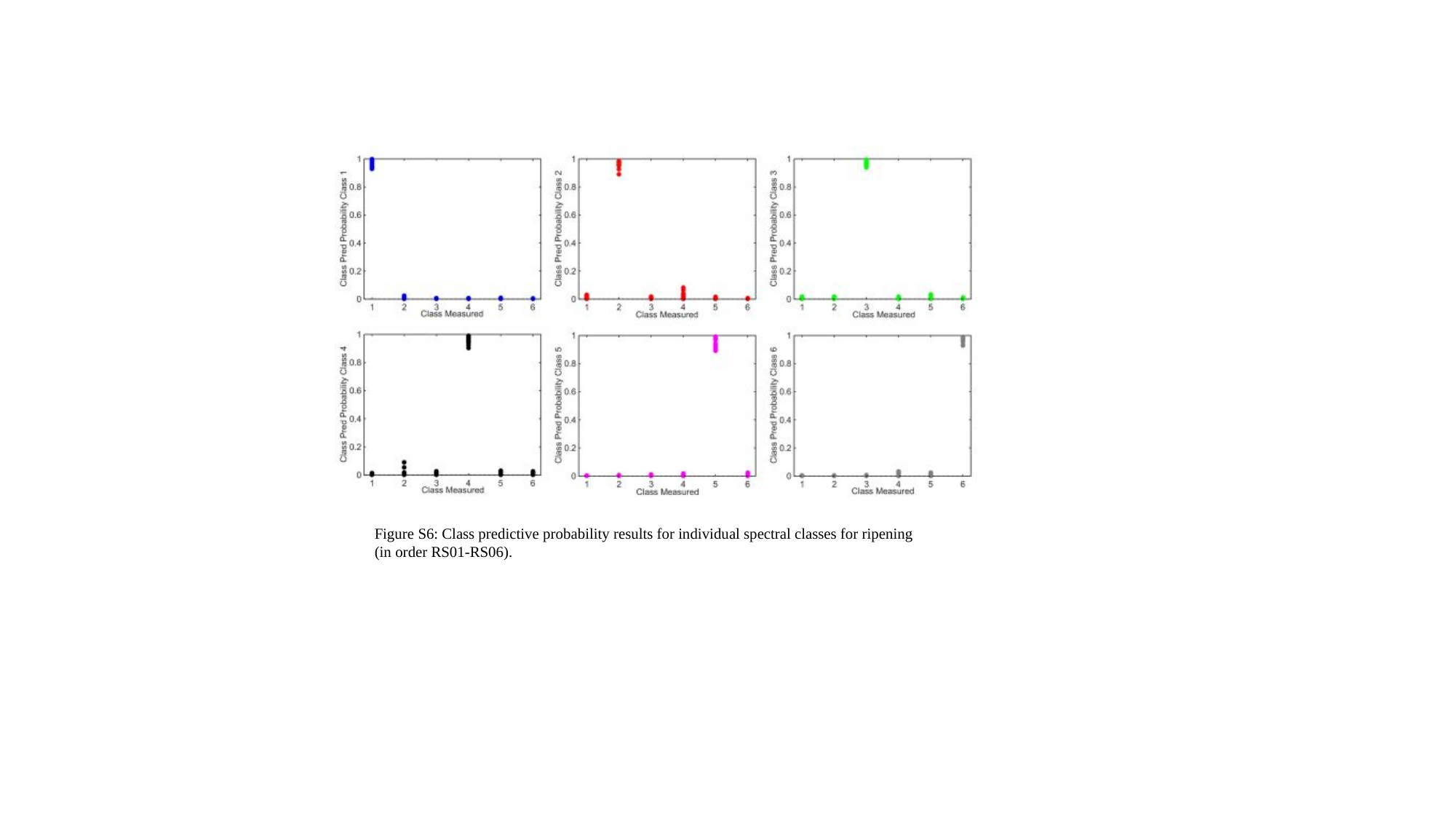

Figure S6: Class predictive probability results for individual spectral classes for ripening
(in order RS01-RS06).
