# Supplementary material for: Determination of developmental and ripening stages of whole tomato fruit using portable infrared spectroscopy and Chemometrics
Source: BMC Plant Biol. 2019 Jun 4;19:236. doi: 10.1186/s12870-019-1852-5 (PMC6549295; doi:10.1186/s12870-019-1852-5)
Supplement: Supplementary file 6 — Table S4. Development stages of tomato fruit S. lycopersicum (cv. Moneymaker), corresponding spectral classes, and their AMS (USDA) grade designation (Kader and Morris [44]; Sargent [43]; Maul et al. [42]). (DOCX 13 kb) [file 12870_2019_1852_MOESM6_ESM.docx]

**Additional File 6**

Table S4: Development stages of tomato fruit *S. lycopersicum* (cv. Moneymaker), corresponding spectral classes, and their AMS (USDA) grade designation (Kader and Morris 1976; Sargent 1996; Maul et al. 1998)

| **Developmental Stage (dpa)** | **Spectral Class** | **Average Weight (grams)** | **Average Diameter (cm)** | **AMS (USDA)**  **Classification** |
| --- | --- | --- | --- | --- |
| 04 | DS01 | 2.8±0.2 | 0.62±0.02 | M-1 |
| 08 | DS02 | 4.9±0.1 | 1.16±0.03 | M-1 |
| 12 | DS03 | 6.8±0.1 | 1.96±0.07 | M-2 |
| 16 | DS04 | 12.8±0.2 | 2.99±0.04 | M-2 |
| 20 | DS05 | 21.5±0.3 | 4.84±0.09 | M-2/M-3 |
| 24 | DS06 | 51.6±0.2 | 5.39±0.08 | M-3 (small) |
| 28 | DS07 | 88.2±0.5 | 6.41±0.13 | M-3 (medium) |
| 32 | DS08 | 145.7±1.0 | 7.01±0.07 | M-3 (large) |
| 36 | DS09 | 176.8±2.3 | 7.71±0.05 | M-4 (extra-large) |
