# Supplementary material for: Determination of developmental and ripening stages of whole tomato fruit using portable infrared spectroscopy and Chemometrics
Source: BMC Plant Biol. 2019 Jun 4;19:236. doi: 10.1186/s12870-019-1852-5 (PMC6549295; doi:10.1186/s12870-019-1852-5)
Supplement: Supplementary file 7 — Table S5. Ripening stages of tomato fruit S. lycopersicum (cv. Moneymaker), corresponding AMS (USDA) ripening and spectral class designation (Sargent [43]; Maul et al. [42]). Fruit used for ripening stages had an average diameter of 7.31 ± 0.24 cm. (DOCX 12 kb) [file 12870_2019_1852_MOESM7_ESM.docx]

**Additional File 7**

Table S5: Ripening stages of tomato fruit *S. lycopersicum* (cv. Moneymaker), corresponding AMS (USDA) ripening and spectral class designation (Sargent 1996; Maul et al. 1998). Fruit used for ripening stages had an average diameter of 7.31±0.24cm.

| **Ripening Stage** | **Spectral Class** | **AMS/USDA Description** |
| --- | --- | --- |
| Mature Green | RS01 | Fruit surface is completely green; shade may vary light to dark |
| Breaker | RS02 | Break in colour from green to tannish-yellow, pink, or red on not more than 10% of the surface colour |
| Turning | RS03 | 10%-30% of the surface is not green; the aggregate shows a definite change from green to tannish-yellow and/or pink/red colour |
| Pink | RS04 | 30%-60% of the surface is not green; the aggregate, shows pink or red colour |
| Light Red | RS05 | 60%-90% of the surface is not green; the aggregate shows pinkish-red or red colour |
| Red (Ripe) | RS06 | > 90% of the surface is not green; aggregate shows red colour |
