# Supplementary material for: Determination of developmental and ripening stages of whole tomato fruit using portable infrared spectroscopy and Chemometrics
Source: BMC Plant Biol. 2019 Jun 4;19:236. doi: 10.1186/s12870-019-1852-5 (PMC6549295; doi:10.1186/s12870-019-1852-5)
Supplement: Supplementary file 8 — Table S6. Percentage of variance for PCA-LDA models varying the number of PCs. (DOCX 17 kb) [file 12870_2019_1852_MOESM8_ESM.docx]

**Additional File 8**

Table S6: Percentage of variance for PCA-LDA models varying the number of PCs.

|  | Developmental Stage (dpa) | | Ripening Stage | |
| --- | --- | --- | --- | --- |
| PC | Variance (%) | Cumulative Variance (%) | Variance (%) | Cumulative Variance (%) |
| 1 | 63.80 | 63.80 | 49.15 | 49.15 |
| 2 | 16.77 | 80.58 | 37.01 | 86.15 |
| 3 | 8.77 | 89.35 | 3.39 | 89.54 |
| 4 | 3.60 | 92.94 | 1.57 | 91.12 |
| 5 | 2.34 | 95.28 | 1.35 | 92.47 |
| 6 | 1.00 | 96.28 | 0.86 | 93.33 |
| 7 | 0.62 | 96.91 | 0.69 | 94.02 |
| 8 | 0.49 | 97.39 | 0.57 | 94.59 |
| 9 | 0.32 | 97.71 | 0.44 | 95.03 |
| 10 | 0.25 | 97.96 | 0.35 | 95.38 |
